# Chemical Composition and, Cellular Evaluation of the Antioxidant Activity of *Desmodium adscendens* Leaves

**DOI:** 10.1155/2011/620862

**Published:** 2010-10-13

**Authors:** François Nsemi Muanda, Jaouad Bouayed, Abdelouaheb Djilani, Chunyan Yao, Rachid Soulimani, Amadou Dicko

**Affiliations:** ^1^Chemistry Laboratory and Methodology for the Environment, Paul-Verlaine University Metz, 1, boulevard Arago Technopole 2000, 57078 Metz, France; ^2^Environment and Agro-Biotechnolgies Department, CRP—Gabriel Lippmann, L-4422 Belvaux, Luxembourg; ^3^Laboratory of Neurotoxicology Alimentary and Bioactivity, Paul-Verlaine University Metz, BP 4102, 57040 Metz, France

## Abstract

*Desmodium adscendens* plant is widely used as juice or tea in various parts of the world against a wide range of diseases. This study determines the quality and the quantity of polyphenols, flavonoids, anthocyanins, and tannins in *D. adscendens* leaves by UV-spectrophotometry and RP-HPLC methods. In addition, the antioxidant capacity of these phenolic compounds is evaluated by ABTS (2,2′-azino-bis(3-ethylbenzothiazoline-6-sulfonic)), DPPH (2,2-diphenyl-1 picrylhydrazyl), and Cellular tests. *D. adscendens* leaves are mainly composite of flavonoid compounds with 12.8 mg of catechin equivalent (CE)/g dw. The amounts of total polyphenol compounds are 11.1 mg of gallic acid equivalent (GAE)/g dw. The quantity of total anthocyanin and total tannin compounds is not considerable 0.0182 mg CgE/g dw and 0.39 mg CE/g dw, respectively. A direct correlation between phenolic compounds and antioxidant activity is observed (*R* ^2^ = 0.96). The RP-HPLC analyses reveal that the main phenolic compound identified in the methanol-water extract is quercetrin dihydrat (2.11 mg/mL). According to the results, it is observed that *D. adscendens* leaves possess a considerable scavenging antioxidant and antiradical capacity, therefore these antioxidant properties might increase the therapeutic value of this medicinal plant.

## 1. Introduction

Medicinal plants are of great importance to the health of individuals and communities; many people in the world have difficulty in gaining access to modern medicine; they use traditional medicine, based on the use of medicinal herbs and plants, as an alternative to a conventional treatment for their recovery. Several papers have been published for pharmacological proprieties in medicinal plants or their isolated constituents like antioxidant, antidiabetic, antibacterial, antiviral, and antiulcer activities [1–5].

Plants (fruits, vegetables, medicinal herbs, etc.) contain a wide variety of free radical scavenging molecules, such as phenolic compounds (e.g., phenolic acids, flavonoids, anthocyanins, and tannins), nitrogen compounds (alkaloids, amines, and betalains), vitamins, terpenoids (including carotenoids), and some other endogenous metabolites, which are rich in antioxidant activity [6, 7]. Epidemiological studies have shown that many of these antioxidant compounds possess anti-inflammatory, antiatherosclerotic, antitumor, antimutagenic, anticarcinogenic, antibacterial, or antiviral activities to a greater or lesser extent [8–10]. The intake of natural antioxidants has been associated with reduced risks of cancer, cardiovascular disease, diabetes, and diseases associated with ageing [10].

Phenolics inhibit carcinogenesis by affecting the molecular events in the initiation, promotion, and progression stages [11]. They modulate the secretion of protein kinases in tumor cell proliferation and induce the expression of anticarcinogenic enzymes or inhibit induction of cancer-promoting enzymes [12–14]. 

Antioxidant activity is a fundamental important property for human life. Many of the biological functions, including antimutagenicity, anticarcinogenicity, and antiaging, among others, originate from this property [8, 11]. 

Recently, Abu Bakar et al. [15] reported that phenolic compounds have potentially beneficial effects on human health by reducing the occurrence of coronary heart disease, age-related eyes diseases, and artherogenic processes. These compounds also have antioxidant and anti-free-radical properties that allow them to quench free radicals in the body [16]. 

Moreover, it was reported that antioxidants with ROS scavenging ability have great relevance in the prevention of oxidative stress which is responsible for the majority of diseases [17].

*Desmodium adscendens* is a vine, which grows wild in the Amazon rainforest of Peru, in South American countries and as well on the West Coast of Africa. Native people use this plant as a juice or a tea. Also, *D. adscendens* is a medicinal plant which is widely used in popular medicine in different parts of the world. In the Brazilian traditional medicine, the leaves of this plant treat leucorrhoea, body aches, pains, ovarian inflammations, excessive urination, gonorrhoea, and diarrhoeas [18]. Its positive effect against hepatic infection was also verified in vivo [19]. In African traditional medicine, *D. adscendens *is extensively used to treat asthma and other diseases associated with smooth muscle contraction [20]. The Congolese healers treat fever, pain, and even epilepsy with aqueous extracts of the leaves of *D. adscendens* [21]. To our knowledge, there is still no report on the phytochemicals and the antioxidant properties of this plant leaf. The present study is aimed to identify, to quantify the phenolic compounds contained in the leaves of *D. adscendens,* and to evaluate their antioxidant capacity by ABTS, DPPH, and cellular tests.

## 2. Materials and Methods

### 2.1. Chemicals

All the chemicals used were of analytical grade. 1,1-Diphenyl-2-picryl hydrazyl radical (DPPH), 2,2′-azino-bis (3-ethylbenzothiazoline-6-sulfonic) (ABTS), HBSS buffer, dioxygen water (H_2_O_2_), 2,2′-azo-bis (2-amidino-propane) dihydrochloride (AAPH), 2′,7′-diacetate dichlorofluorescein (DCFH-DA), gallic acid, folin-Ciocalteu's phenol reagent, alumin chloride, catechin, *p*-coumaric acid, rutin, procatechiuc acid, vitamin C, caffeic acid, isovitexin, vitexin, chlorogenic acid, orientin, malvidin, homoorientin, peonidin, catechin, quercetin, quercetin dihydrat, quercetin-3-*β*-D-glucosyl, epicatechin, kuromanin chloride, and cyanidin chloride were purchased from Across organics (Geel, Belgium). Sodium carbonate, sodium nitrite, chlorhydric acid, ethyl acetate sodium, sodium sulphate anhydrous, ammonium phosphate, ferric ammonium sulphate, acetonitrile, methanol, vanillin reagent, and n-hexane were obtained from Sigma and Roth (Strasbourg, France).

### 2.2. Apparatus

The RP-HPLC analyses were performed with a Waters 600E pump coupled to a Waters 486 UV visible tunable detector and equipped with a 20 *μ*L injection loop and an Alltech Intertsil ODS column (RP C18 column size 4.6 mm × 150 mm; particle size, 5 *μ*m). 

Flow cytometry is used to separate the different immune cell populations according to size (forward light scatter, FSC) and relative granularity (side light scatter, SSC). FSC and SSC were used after excitation of the immune cells with using 488 nm argon laser beams. The level of intracellular ROS was measured in the granulocytes by monitoring the emitted fluorescence of these cells (FACS-Scan, Becton-Dickinson, Immunofluorometry Systems, France). In addition, spectrophotometer analyses were carried out with UV-Vis spectrophotometer (Cary 50 scan).

### 2.3. Animals

Swiss albino male mice (OF1) aged 9 weeks at the receipt from the breeder company (Charles River, France) and weighing 40–45 g were used. They were maintained under standard environmental conditions with free access to water and food (SD Dieted-France). All animal procedures were carried out in accordance with the European Communities Council Directive of 24 November 1986 (86/609/EEC).

### 2.4. Plant Materials

Dried leaves of *D. adscendens *(Fabaceae) were obtained from Nigeria, and the biological authentication was carried out by Professor Max Henry in the Botanic and Mycology Laboratory, Nancy University (France). All plant materials were dried at room temperature and were ground and sifted in a sieve (0.75 *μ*m).

### 2.5. Sample Preparation

#### 2.5.1. Total Phenolic, Flavonoid, and Anthocyanin Contents and Antioxidant Capacity

Samples for total phenolic compounds, total flavonoid compounds, total anthocyanin compounds, and antioxidant capacity assays were extracted from the powders as described by Chitindingue et al. [16]. 

Two grams of powdered sample were extracted twice with 10 mL of cold aqueous methanol solution (50%). The two volumes were combined, made up to 40 mL, centrifuged at 1536 × g for 20 minutes, transferred in small sample bottles, and stored at +4°C in the dark until analysis.

### 2.6. Condensed Tannin (CT) Extraction

Samples for CT were extracted from the powders as described by Villarreal-Lozoya et al. [22]. One gram of powder was extracted twice with 20 mL of n-hexane for 20 minutes and filtered, and the remaining powder was dried at 35°C under vacuum for 2 h. The powder was stored at +4°C until analyses.

### 2.7. Phenolic Compounds Extraction for RP-HPLC Analysis

Polyphenols were extracted with water (WE) and with methanol-water (50-50, v-v) (MWE) as solvents according to the slightly modified method described by Sharma et al. [23]. A sample (0.4 g) was extracted with 2 × 5 mL of solvent under intermittent shaking (2 minutes) on vortex mixer for 30 minutes. The sample was centrifuged at 1536 × g for 20 minutes at 20°C. The supernatant was taken into a 10 mL volumetric flask. The extracts are stable for 24 h if stored at 4°C.

### 2.8. Preparation of Samples for Cellular Tests

The extract was prepared from the dried plant in the traditional way (Nigeria). A decoction was done for 1 h in distilled boiled (200 g/L) water. Then the resulting mixture was filtered and evaporated under reduced pressure and lyophilized by a Lyophilizer (ALPHA 1-2 LD, Fisher Bio Block). The amount of lyophilized extract obtained was 24.2 g.

### 2.9. Dosage of Phenolic Compounds

#### 2.9.1. Total Phenolic Compounds

The Folin-Ciocalteu method was used to measure the total phenolic compounds (Dzingirai et al. [4]). To a sample (100 *μ*L), distilled water was added to make 2 mL (Eppendorff tube), followed by 1 mL of Folin Ciocalteu reagent 1 N and sodium carbonate (20%). 

After 40 minutes at room temperature, absorbance at 725 nm was read on a Spectrophotometer against a blank that contained methanol instead to sample. The results were compared to a gallic acid calibration curve, and the total phenolic compounds were determined as gallic acid equivalents (GAEs). The samples were analyzed at least three replications.

#### 2.9.2. Total Flavonoid Compounds

The flavonoid contents were measured according to a colorimetric assay reported by Muanda et al. [24]. A 250 *μ*L of standard solution of catechin at different concentrations or appropriately diluted samples was added to 10 mL volumetric flask containing 1 mL of distillate water. At the initial time, 75 *μ*L of NaNO_2_ (5%) was added to the flask. After 5 minutes, 75 *μ*L of AlCl_3_ (10%) was added and after 6 minutes, 500 *μ*L of NaOH (1 N) was added to mixture. Immediately, the solution was diluted by adding 2.5 mL of distillate water and mixed thoroughly. Absorbance of the mixture, pink in colour, was determined at 510 nm versus the prepared blank. Total flavonoid compounds in medicinal plant were expressed as catechin equivalents (CEs). Samples were analysed in three replications.

#### 2.9.3. Total Anthocyanin Compounds

The total anthocyanin compound of the samples was estimated using a UV-spectrophotometer by the pH-differential method [15, 25]. Two buffer systems, potassium chloride buffer, pH 1.0 (0.025 M) and sodium acetate buffer, pH 4.5 (0.4 M) were used. Briefly, 400 *μ*L of extract was mixed in 3.6 mL of corresponding buffer solutions and read against a blank at 510 and 700 nm. Absorbance (ΔA) was calculated as ΔA = (A_510_ − A_700_)pH  1.0 − (A_510_ − A_700_) pH 4.5, monomeric anthocyanin pigment concentration in the extract was calculated and expressed as equivalent cyaniding-3-glucoside (mg/L): ΔA × Mw × Df × 1000/(Ma × 1), with ΔA: difference of absorbance, Mw: molecular weight for cyaniding-3-glucoside (449.2), Df: the dilution factor of the samples, and Ma: molar absorptivity of cyaniding-3-glucoside (26 900). Results were expressed as mg of Cyaniding-3-glucoside Equivalents (CgEs).

#### 2.9.4. Condensed Tannins

The condensed tannin content was estimated using the method described by Villarreal-Lozoya et al. [22] with some modifications. Briefly, an aliquot of 0.5 g of powder obtained after lixiviation (n-hexane) was placed in a centrifuge tube and 15 mL of 1% HCl in methanol was added to each sample. Each tube was vortexed and placed in a water bath at 35°C with constant shaking for 20 minutes and vortexing every 5 minutes. After incubation, the tubes were centrifuged (1536 × g), and the supernatants were extracted. Aliquots of the supernatants (100 *μ*L) were placed in two separate assay tubes, one for the sample determination and the other for blank determination.

Samples and blanks were incubated for exactly 20 minutes after adding 5 mL of vanillin reagent (0.5 g of reagent and 200 mL of 4% HCl methanol) to samples and 4% HCl in methanol to the blanks. After 20 minutes, absorbance was read at 500 nm from each sample and blank using UV-spectrophotometer Varian Cary 50. Samples absorbance was rectified with the blank standard and compared against a standard curve made with catechin. Results were expressed as mg catechin equivalent (CE) of lixiviating sample. Analyses were triplicate.

### 2.10. RP-HPLC Analysis

The samples (WE) and (MWE) were filtered through a 0.45 *μ*m PTFE syringe tip filter and were analyzed using an RP-HPLC system equipped with a 20 *μ*L injection loop, a waters UV-Visible tuneable detector on a reverse phase (RP C_18_) column Alltech Interstsil ODS-5 *μ*m × 4.6 mm × 150 mm. The flow rate was set at 1 mL/minute at room temperature. To perform this study, a gradient of three mobile phases was used; solvent A: 50 mM ammonium phosphate (NH_4_H_2_PO_4_) pH 2.6 (adjusted with phosphoric acid), solvent B: (80 : 20 (v/v)) acetonitrile/solvent A, and solvent C: 200 mM of phosphoric acid pH 1.5 (pH adjusted with ammonium hydroxide). The solvents were filtered through a Whatman Maidstone England paper N° 3 and placed in an ultrasonic apparatus for 25 minutes. The gradient profile was linearly changed as follows (total 60 minutes): 100% solvent A at zero minutes, 92% A/8% B at 4 minutes, 14% B/86% C at 10 minutes, 16% B/84% C at 22.5 minutes, 25% B/75% C at 27.5 minutes, 80% B/20% C at 50 minutes, 100% solvent A at 55 minutes, and 100% A at 60 minutes. 

After each run, the system was reconditioned for 10 minutes before analysis of the next sample. Under these conditions, each sample analysis was done in triplicate. Polyphenolic external standards were prepared by dissolving 2 mg/mL and used as reference. In each sample, polyphenol was identified by comparing its retention time with that of the corresponding external standard. Detection was done at 280 and 320 nm, and quantification was calculated by comparing their peak areas.

### 2.11. Antioxidant Activity Analyses

#### 2.11.1. DPPH Radical Scavenging Test

The DPPH radical scavenging activity was evaluated according to the method slightly modified described by Marwah et al., Sharififar et al., Adesengun et al., and Al-Zubairi et al. [26–29]. 1 mL of 100 *μ*M DPPH solution in methanol was mixed with 1 mL of plant extract. The reaction mixture was incubated in the dark for 20 minutes, and the optical density was recorded at 517 nm against the blank. For the control, 1 mL of DPPH solution in methanol was mixed with 1 mL of methanol, and optical density of solution was recorded after 20 minutes.

The decrease in optical density of DPPH in samples with regard to control system was used to calculate the antioxidant activity as a percentage inhibition (IP%) of DPPH radical, IP% = ((A_to_ − At_20_)/(A_to_ × 100)) were A_to_: absorbance of the test sample after zero minutes and At_2o_: absorbance of control after 20 minutes. Each assay was carried out in triplicate. 

From a plot of concentration against %IP, a linear regression analysis was performed to determine the IC_50_ value for each extract. The DPPH radical scavenging activity of phenolic compounds was expressed as IC_50_ value in terms of *μ*g/mL of dry weight. To 2.90 mL of an aqueous methanol solution (50%) of 100 *μ*M of DPPH, 100 *μ*L of the plant extract solution was added. The mixture was shaken and stored at 20°C in the dark for 40 minutes. Then, the decrease of absorbance was measured; the resulting solution was monitored at 517 nm. The DPPH radical scavenging activity of phenolic compounds was expressed in terms of mg of Vitamin C Equivalents (VCEs) per g dw in 40 minutes. The control solution was consisted of 100 *μ*L of methanol and 2.90 mL of DPPH solution. The radical solution was prepared daily.

#### 2.11.2. ABTS Radical Scavenging Test

The slightly modified method developed by Djeridane et al. [30] was used in this experiment. 1.0 mM of AAPH solution was mixed with 2.5 mM ABTS as diammonium salt in phosphate buffered saline (PBS) solution 100 mM potassium phosphate buffered (pH 7.4) containing 150 mM NaCl. The mixture was heated in a water bath at 68°C for 20 minutes. The concentration of the resulting blue-green ABTS^•+^ (radical cation solution) was adjusted to an absorbance of 0.65 ± 0.02 at 734 nm. 

The sample solution of 60 *μ*L was added to 2.94 mL of the resulting blue-green ABTS radical solution. The mixture, protected from light, was incubated in a water bath at 37°C for 20 minutes.

Then the decrease of the absorbance at 734 nm was measured. The control solution was consisted of 60 *μ*L of methanol and 2.94 mL of ABTS^•+^ solution. The stable ABTS radical cation scavenging activity of the phenolic compounds in the extract was expressed in terms of mg of Vitamin C Equivalents (VCEs) per g dw in 20 minutes. All radical stock solutions were prepared daily.

#### 2.11.3. Cellular Test

 A cellular assay was used for the in vitro antioxidant activity evaluation; flow cytometry technique allows a separation of different immune cells population by size (forward light scatter, FSC) and relative granularity (side light scatter, ssc) parameters. FSC and SSC were used after excitation of the immune cells by the 488 nm laser beam of argon. The level of intracellular ROS was measured in the granulocytes by monitoring of the emitted fluorescence by these cells.

The ROS level was quantified using 2′,7′-diacetate dichlorofluorescein (DCFH-DA). Nonpolar DCFH-DA crosses the cellular membranes and is hydrolysed by intracellular esterase to form the polar, nonfluorescent dichlorofluorescein (DCFH). Then, the latter is oxidized to highly fluorescent substance, the 2′,7′-dichorofluorescein (DCF) by intracellular ROS [31, 32]. 

The mice were anesthetized with the halothane and sacrificed. The collected blood was heparinized and dispatched in volumes of 100 *μ*L in Eppendorff tubes. Erythrose was done using 2 mL of lysing solution in each tube; the whole was placed in darkness for 10 minutes. After centrifugation at 4°C (5 min; 1536 × g), the supernatant was eliminated, then 2 mL cell wash solution was added to sediment containing white cells, mixed, and followed by a new centrifugation in the same conditions. Supernatant was still eliminated.

Three groups of cell belonging to the same blood were used to assess the intracellular ROS level; the first one was a control without oxidative stress, and the second was a control with oxidative stress. The third group is served to evaluate the antioxidant activity of the extract. 1 mL of HBSS buffer was added and 5 *μ*L of DCFH-DA (50 *μ*M) to white cells. For the control (without oxidative stress), incubation was achieved in darkness during 30 minutes at 37°C. For the second control (with oxidative stress), incubation was performed in darkness during 15 min at 37°C, then is added 5 *μ*L of H_2_O_2_ (89 mM) to provoke oxidative stress, the incubation continued for 15 minutes more.

In the third group of cells, several concentrations of the extract (*D. adcendens*) were used for the evaluation of its antioxidant activity. So, to this group, in addition to HBSS buffer and DCFH-DA, 5 *μ*L of *D. adscendens *extract were added. After 15 minutes of incubation in darkness, the oxidative stress was induced by addition of 5 *μ*L of H_2_O_2_ (89 mM). It continued for 15 minutes at 37°C. As soon as incubation times were completed, the ROS levels were measured by the flow cytometry technique.

In this test, oxidative stress is induced by addition of H_2_O_2_ in the extracellular medium of the granulocytes. Exogenous H_2_O_2_ diffuses inside the cells, consequently their DCFH oxidation increases (C) compared to granulocytes without oxidative stress (A). Under oxidative stress conditions, *D. adscendens *extract led to the reduction of the fluorescence intensity of treated cells (B). The fluorescence intensity is expressed as decimal logarithm versus the cell number. (A) and (B) are the controls corresponding to the level of intracellular ROS without and with oxidative stress, respectively. (C) is the treated granulocytes corresponding to ROS level in these cells incubated in the presence of *D. adscendens *extract at 25 mg/mL then subjected to the oxidative stress.

The reduction percentage of ROS generated in granulocytes by exogenous H_2_O_2_ was calculated by the monitoring of the emitted fluorescence intensity (Fi). The following relation was used (Fit_o_ − Fit_1_) × 100/(Fit_o_ − Fit_2_) with Fit_o_: control with oxidative stress; Fit_1_: treat cells; Fit_2_: control without oxidative stress.

### 2.12. Statistical Analysis

Results are presented as mean ± standard Error; statistical analysis of experimental result was based on analysis of variance. Observed differences were statistically considered significant at the level of *P* < .001.

## 3. Results

### 3.1. Total Phenolic Compounds

**Figure 1:**
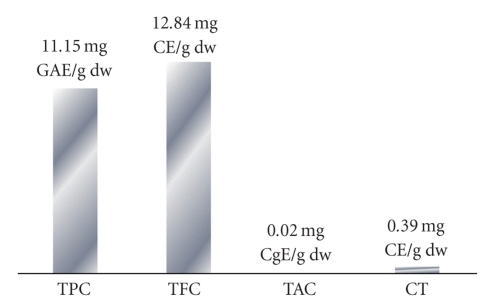
Phenolic compounds of leaves of *D. adscendens*. TPC: Total phenolic compounds; TFC: Total flavonoid Compounds; TAC: Total anthocyanin compounds; CT: Condensed tannins; dw: dry weight; GAE: Gallic Acid Equivalent; CE: Catechin Equivalent; CgE: Cyaniding-3-glycoside Equivalent.

The results reveal that 1 g of dry weight of *D. adscendens* leaves contains 11.15 mg GAE of total phenolic compounds, 12.84 mg CE of total flavonoid compounds, and 0.0182 mg CgE of total anthocyanins, and the amount of condensed tannins observed is 0.39 mg CE (Figure 1).

### 3.2. HPLC Analysis

**Figure 2:**
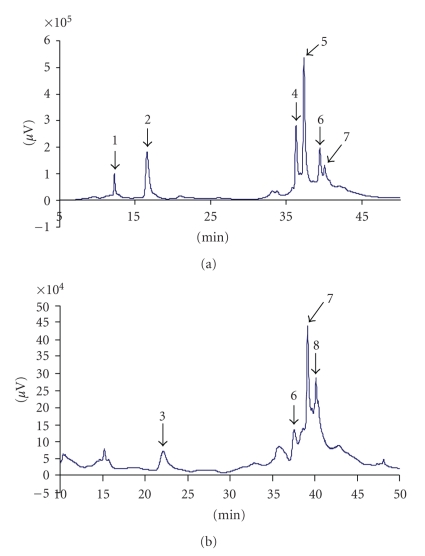
(a) WE, HPLC chromatogram at 280 nm of the leaves of *D. adscendens* 1: gallic acid; 2: protocatechuic acid; 4: rutin; 5: quercetrin glucosyl; 6: quercetrin dihydrat; 7: cinnamic acid. (b) WE, HPLC chromatogram at 320 nm of the leaves of *D. adscendens* 3: Catechin; 6: quercetrin glucosyl; 7: quercetrin dihydrat; 8: cinnamic acid.

**Figure 3:**
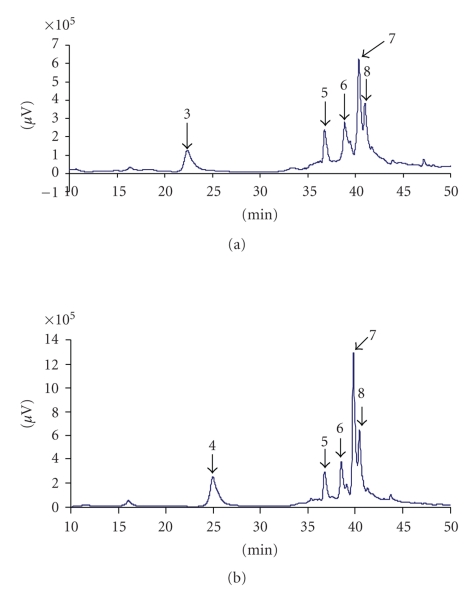
(a) MWE, HPLC chromatogram at 280 nm of leaves of *D. adscendens* 3: catechin; 6: quercetrin glucosyl; 7: quercetrin dihydrat; 8: cinnamic acid. (b) MWE, HPLC chromatogram at 320 nm of leaves of *D. adscendens* 4: chlorogenic acid; 5: quercetrin glycosyl; 6: quercetrin dihydrat; 7: cinnamic acid; 8: cinnamic acid.

The RP-HPLC profiles of the water extracts and the methanol-water extracts are shown in Figures 2 and 3, respectively. Peaks were identified and quantified on the basis of their retention time values and UV spectra by comparison with those of the single compound in the standard solution. The retention time and the concentration of polyphenolic compounds contained in the extracts are reported in Table 1. There were numerous peaks that were not identified because of the lack of suitable standards. The samples were analyzed at least four replications at 280 and 320 nm.

### 3.3. Antioxidants Activities

#### 3.3.1. ABTS and DPPH Tests

The ABTS test and the DPPH test reveal that the extracts of *D. adscendens* leaves possess a useful scavenging antioxidant activities, these results, expressed as mg of VCE/g dw, are 12.83 mg (ABTS) and 8.47 mg DPPH.

#### 3.3.2. Cellular Test

**Figure 4:**
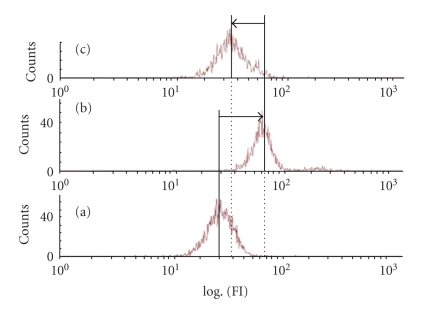
The ROS levels were measured by flow cytometry. The fluorescence intensity was expressed as decimal logarithm versus the cell number. (a) and (b) are the controls corresponding to the level of intracellular ROS without and with oxidative stress, respectively. (c) is the pretreated granulocytes corresponding to ROS level in these cells incubated in the presence of *Desmodium adscendens* extract at 25 mg/mL then subjected to the oxidative stress.

The ROS level was detected using a fluorescence probe, 2′,7′-dichlorofluorescin diacetate (DCFH-DA), which can be oxidized to highly fluorescent dichlorofluorescein (DCF) by intracellular ROS [16]. In Figure 4, a decrease of the fluorescence intensity (FI) was observed, between (B) (log = 80) corresponding to control with oxidative stress and (C) (log = 35) representing the pretreated cells, which was close to the level of the control of cells without oxidative stress (A) (log = 30). This decrease is due to the effect of the extract of *D. adscendens *on the ROS level which is directly correlated to the FI. 

The scavenging capacity of* D*. *adscendens leaves* extract on the ROS was highlighted. To a concentration of 25 mg/mL, it is observed a reduction of 83.21 ± 6.21% of ROS level generated by exogenous H_2_O_2_. Thus, pretreatment of granulocytes with this extract appreciably inhibited the ROS generation induced by H_2_O_2_.

## 4. Discussion

Among the phenolic compounds contained in the leaves of *D. adscendens,* the major was the anthocyanins, and the least was the condensed tannins.

Quantitative analyses carried out by RP-HPLC show that water is not the best extract solvent for polyphenolic compounds. This is in concordance with Marwah et al. [26], who have reported that aqueous alcohols are the best solvents for extracting polyphenolic compounds from plant materials. However, these results show that, in water, gallic acid (0.12 mg/mL) and the protocatechuic acid (0.11 mg/mL) are more extracted than in the methanol-water medium because these acids can be transformed into their corresponding esters in hydroalcoholic medium [33]. 

The major compound identified in the WE was the quercetrin glucosyl (0.46 mg/mL) and the least was catechin (0.06 mg/mL). 

In contrast, it was found that, in the MWE, the quercetrin glucosyl (0.17 mg/mL) was the least. In this extract, the quercetrin dihydrat concentration (2.11 mg/mL) was the highest followed by cinnamic acid (0.76 mg/mL). 

The antioxidant activities recorded both using the ABTS test and the DPPH test show that *D. ascendens *leaves possess useful antioxidant properties. To compare these activities with those other medicinal plants previously described in the literature such as* Allophylus rubifolius, Phaulopsis fascisepala, Anogeissus dhofarica, *and* Litchi* seeds, the antioxidant activities were expressed as IC_50_. The IC_50_ value of *D. adscendens* extract was 4.00 *μ*g/mL; this result reveals that compared to* Allophylus rubifolius* (IC_50_ = 7.1 *μ*g/mL) [26], *Phaulopsis fascisepala* leaves (IC_50_ = 0.5 mg/mL) [28], *Anogeissus dhofarica* (IC_50_ = 4.5 *μ*g/mL), and* Litchi* seeds (IC_50_ = 4.8 *μ*g/mL) [34], *D. adscendens* leaves possess a significant antioxidant activities, since more the value of IC_50_ is low more the antioxidant activity is high.

**Figure 5:**
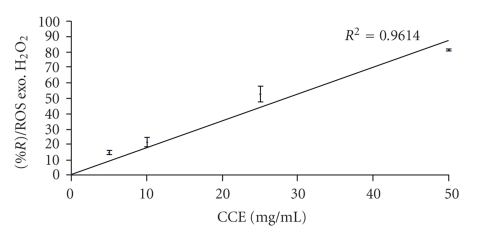
Concentration-response curve for reduction of ROS generated by exogenous H_2_O_2_. *R* (%) ROS exo H_2_O_2_, reduction (%) of ROS generated by exogenous H_2_O_2_; CCE: Concentration of extract.

In order to confirm these results, the antioxidant capacity of *D. adscendens *was evaluated against ROS under biological medium by using a cellular test. The DCFH-DA was used to evaluate the intracellular redox status [28]. Concentration-response curve in the ROS reduction reveals a linear and positive relationship (*R* ^2^ = 0.96) between the scavenger capacity and the concentration of *D. adscendens *extracts. Then, this reduction is directly correlated to the ROS level decreasing (Figure 5). 

The ROS are high energy forms of oxygen, they induce hyperoxidation, cytotoxicity of oxygen and they decrease the antioxidant activity [35, 36]. The present study indicates that *D. adscendens* extract restrains scavenging activity of ROS.

The reducing capacity of extracts of *D. adscendens* leaves may be used as a significant indicator of its potential antioxidant activity. As shown in Figures 4 and 5, it is observed that the plant extract has some reducing capacity, justifying its antioxidant properties.

Comparing to some plant extracts from *Ipomea batata, Moringa oleifera, Albelmoshus manihot*, *Latuca sativa* [25], *Oudneya africana, Thapsia garganica, Thymelaea hirsute, Teucrium polium, Artemisia arborescens, Ruta montana* [30], *D. adscendens* plant extract shows significant amount of phenolic, flavonoid, anthocyanin, and tannin compounds with antioxidant properties. 

The antioxidative properties have been demonstrated by several studies in the literature [24, 36]. Polyphenolic, flavonoid, anthocyanin, and tannin compounds have high antioxidant properties, and their effects are significant on human nutrition and health. It has been reported that the body's antioxidant defense system consists of the activity of SOD, CAT, GST, and GSH [37]. SOD catalyzes the breakdown of endogenous cytotoxic superoxide radicals to H_2_O_2_ which is further degraded by CAT. Thus, they play a crucial role in maintaining the physiological levels of O_2_ and H_2_O_2_. GSH, in conjunction with GST, has a basic role in cellular defense against deleterious free radicals and other oxidant species [38]. GST catalyzes the conjugation of thiol group of glutathione to electrophilic substrates and thereby detoxifies endogenous compounds such as peroxidized lipids [39]. 

**Figure 6:**
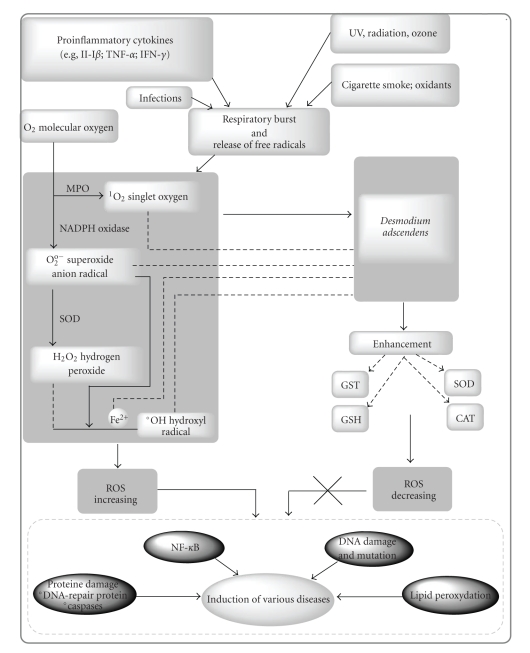
Role of *D. adcendens* in prevention of diseases caused by free radicals. Generation of ROS is initiated by respiratory burst, which is set off by various physiological and environmental factors. The fabrication of an assortment of ROS from the molecular O_2_, carried by different enzymes such as MPO (myloperoxidase), NADPH oxidase, and SOD (superoxide dismutase), leads to diverse cellular phenomena, namely, damage of DNA-repair proteins and caspases, lipid peroxydation, and DNA damage followed by mutation and NF-*κ*B activation. All these phenomena give rise to wide range of diseases. *D. adscendens* leaves extract inhibits the generations of the free radicals by scavenging both the mother and the daughter products and also by inducing the increase of SOD, CAT, GST, and GSH, resulting in the obstruction of various disease formation.

Our results signify that this plant is an important source of natural antioxidant, which could play a vital role in preventing the progress of various oxidative stresses, in the course of enhancing the generation of typical antioxidant enzymes (Figure 6) as illustrated by Mandal et al. [17], which involved the NO^•^ radical in the process. 

In conclusion, the aim of the present study was to evaluate the antioxidant potency of the *D. adscendens* plant extract and to determine the polyphenolic compound contents. Since the extracts of *D. adscendens* leaves exhibit interesting antioxidant properties and ROS scavenging activity, the *D. adscendens* may be considered a rich source of natural antioxidants, which justifies its use in folk medicine. Furthermore, evaluation of in vitro antioxidant activity of these extracts has also provided interesting results that might be beneficial for the pharmacological use of this plant in clinical trials.

## Figures and Tables

**Table 1:** Concentration of the phenolic compounds in the WE and the MWE.

Rt (min)	Ref.	Name of compounds	WE (mg mL^−1^)	MWE (mg mL^−1^)
11.98 ± 0.22	[1]	Gallic acid	0.12 ± 0.01	N.d
14.78 ± 0.92	[2]	Protocatechiuc acid	0.11 ± 0.02	N.d
23.96 ± 0.23	[3]	Catechin	0.06 ± 0.04	0.69 ± 0.04
25.80 ± 0.23	[4]	Chlorogenic acid	N.d	0.23 ± 0.06
37.02 ± 0.36	[5]	Rutin	0.24 ± 0.01	0.28 ± 0.04
37.71 ± 0.18	[6]	Quercitin glucosyl	0.46 ± 0.03	0.17 ± 0.01
39.47 ± 0.19	[7]	Quercitin dihydrat	0.17 ± 0.02	2.11 ± 0.16
41.68 ± 0.65	[8]	Cinnamic acid	0.14 ± 0.01	0.76 ± 0.07

WE: water extract; MWE: methanol water extract; Rt: Retention time in minute; N.d: not detected.
